# Repellents Inhibit P450 Enzymes in *Stegomyia (Aedes) aegypti*

**DOI:** 10.1371/journal.pone.0048698

**Published:** 2012-11-13

**Authors:** Gloria Isabel Jaramillo Ramirez, James G. Logan, Elisa Loza-Reyes, Elena Stashenko, Graham D. Moores

**Affiliations:** 1 Department of Biological Chemistry, Rothamsted Research, Harpenden, United Kingdom; 2 Centro Internacional de Entrenamiento e Investigaciones Médicas, Cali, Colombia; 3 Department of Disease Control, London School of Hygiene and Tropical Medicine, London, United Kingdom; 4 Department of Computational and Systems Biology, Rothamsted Research, Harpenden, United Kingdom; 5 Centro Nacional de Investigaciones para la Agroindustrialización de Especies Vegetales Aromáticas Medicinales Tropicales, Bucaramanga, Colombia; 6 ApresLabs Ltd, Rothamsted Research, Harpenden, United Kingdom; University of Crete, Greece

## Abstract

The primary defence against mosquitoes and other disease vectors is often the application of a repellent. Despite their common use, the mechanism(s) underlying the activity of repellents is not fully understood, with even the mode of action of DEET having been reported to be via different mechanisms; e.g. interference with olfactory receptor neurones or actively detected by olfactory receptor neurones on the antennae or maxillary palps. In this study, we discuss a novel mechanism for repellence, one of P450 inhibition. Thirteen essential oil extracts from Colombian plants were assayed for potency as P450 inhibitors, using a kinetic fluorometric assay, and for repellency using a modified World Health Organisation Pesticide Evaluations Scheme (WHOPES) arm-in cage assay with *Stegomyia (Aedes) aegypti* mosquitoes. Bootstrap analysis on the inhibition analysis revealed a significant correlation between P450-inhibition and repellent activity of the oils.

## Introduction

Mosquitoes are of high public concern due to their ability to vector pathogens that cause disease in human beings and the skin reactions caused by their bites. Generally, for the prevention of any arthropod bite, the initial defence is often the application of a repellent [1], [2]. Despite their common use, the mechanism(s) underlying the activity of repellents is not fully understood. An insect repellent is defined as a chemical that produces oriented movements away from its source [3], however, the term “repellent” is often used loosely to describe an active ingredient that prevents biting. Thus, a mechanism that nullifies the attraction of an insect to a source would also be considered a repellent.

Most commercially available repellents are based on DEET (*N,N*-diethyl-m-methylbenzamide). DEET is often considered to be the “gold standard” repellent, being the most widely used and effective repellent, with some manufacturers claiming varying levels of protection for up to 12 hours [4], [5]. The mode of action of DEET has been reported to be via different mechanisms. For example, several studies have shown that DEET can interfere with olfactory receptor neurones that are tuned into detecting semiochemicals that induce and facilitate host seeking behaviour in mosquitoes [6], [7]. Other studies have shown that DEET is actively detected by olfactory receptor neurones on the antennae or maxillary palps [8], [5]. These studies highlight the fact that mosquito repellents may involve more than one mode of action.

DEET has had an excellent safety record since its commercial release in 1956 [9]. However, there have been some health concerns about long term usage, it can have an unpleasant odour, and may be damaging to some plastics and other synthetic materials. The possible adverse properties of DEET have resulted in considerable interest in developing benign natural products as an alternative.

Essential oils (EOs), complex mixtures of volatile compounds isolated from plants, can act as repellents against various haematophagous arthropods and some form the basis of commercial repellent formulations [10], [11]. Various plants have also been found to contain ‘cocktails’ of chemicals with unique biological activity which could act as repellents [12]. As an example, an effective alternative to DEET are formulations containing P-menthane diol (PMD), derived from extracts of lemon eucalyptus [13]. Although there is evidence that some individual compounds within the complex mixture of essential oils can be detected by the antennae of mosquitoes which may contribute to repellency [14], [15], the exact mode of action of essential oils has not been studied in great detail.

The search for natural compounds to replace or complement DEET is, however, made difficult by the lack of understanding as to repellents’ mode of action. Without this knowledge, high-throughput testing is not possible, and lengthy screening methods have to be employed. Although some *in vitro* methods are in development for the identification of new repellents that act through detecting those that interfere with the activity of attractants [16], this process can be complicated and other repellents, with alternative modes of action, may be missed using such a screening system.

Recently, it was found that DEET acted as a synergist, increasing the efficacy of propoxur against mosquitoes, probably acting as a P450 inhibitor [17]. Furthermore, MGK 264, a chemical that was developed as an insecticide synergist, increasing efficacy of insecticides by inhibiting P450 enzymes that had the ability to metabolise natural pyrethrum, was also found to increase efficacy in many repellent mixtures [18]. However, there are no reports concerning a direct relationship between P450 inhibition and repellency, causal or otherwise. The aim of this study was to determine whether such a relationship exists.

## Results and Discussion

A ‘pilot’ study was conducted to determine the P450-inhibitory potency of known repellents using rabbit liver as a source of P450 activity. In this experiment, a single concentration of the repellents was incubated with rabbit liver P450s for 10 minutes and the inhibition assessed. It was found that all the chemicals (except the control-ethanol) inhibited P450 activity by some degree. Geranylacetone was found to be the most potent inhibitor inhibiting activity by 97%. This was followed by DEET (60%), 6-methyl-5-heptene-2-one (60%) and 1-octen-3-ol (59%). These chemicals are known to induce a behavioural response in insects. For example, geranylacetone and 6-methyl-5-heptene-2-one were found to be the most potent repellents from a series of human-derived semiochemicals tested against *S. aegypti* mosquitoes [19]. 1-Octen-3-ol is a known attractant for some species, but it has also been demonstrated to have a repellent effect, particularly at high concentrations [20], [21],22].

The correlation found in this preliminary assay prompted a larger study assessing correlations between the repellent activity and P450-inhibition of thirteen essential oil samples extracted from Colombian plants (Table 1). Extracts having the most potent P450 inhibitors were found to be from the Verbenaceae family mainly, with the three most potent inhibitors being *Lippia origanoides* species. The only member of this species that did not rank highly was *Lippia origanoides* VEsaWCR-01 (Table 2).

**Table 1:** Botanical derivation of essential oils.

Species	Family	Code	Location (city/province)
*Achyrocline olata*	Asteraceae	ATnaW02B	Potosí/Nariño
*Condylidium cuatrecosasii*	Asteraceae	ATsaW13B	Piedecuesta/Santander
*Hyptis mutabilis*	Lamiaceae	LMmeW02H	Villavicencio/Meta
*Lepechinia betonicifolia*	Lamiaceae	LTcuW24E	Bogotá/Cundinamarca
*Lepechinia schiedeana*	Lamiaceae	LBbgW01E	Bucaramanga/Santander
*Ocinum campechianum*	Lamiaceae	LMsuW02B	Tolú Viejo/Sucre
*Lippia alba*	Verbenaceae	VEboW02E	Colorado/Bolívar
*Lippia alba*	Verbenaceae	VEbgW01E	Bucaramanga/Santander
*Lippia origanoides*	Verbenaceae	VEbyW06B	Soatá/Boyacá
*Lippia origanoides*	Verbenaceae	VenaW02B	Pedregal/Nariño
*Lippia origanoides*	Verbenaceae	VEsaWCR-01	Bucaramanga/Santander
*Lippia origanoides*	Verbenaceae	VEsaWCR-02	Bucaramanga/Santander
*Montanoa ovalifolia*	Rivularaceae	ATbyW02L	Tibasosa/Boyacá

**Table 2:** Estimates of mean percentage activity remaining of P450 enzymes after treatment with the 13 essential oils.

Sample	Code	Mean percentage activity remaining (95% CI)
*Lippia origanoides*	VEnaW02B	21.93 (14.474, 33.220)
*Lippia origanoides*	VEbyW06B	26.18 (17.282, 39.664)
*Lippia origanoides*	VEsaWCR-02	26.00 (17.163, 39.391)
*Lippia alba*	VEbgW01E	30.20 (19.934, 45.751)
*Condylidium cuatrecosasii*	ATsaW13B	31.19 (20.587, 47.250)
*Lippia alba*	VEboW02E	33.73 (22.264, 51.098)
*Ocinum campechianum*	LMsuW02B	39.36 (25.978, 59.621)
*Lepechinia betonicifolia*	LTcuW24E	40.55 (26.767, 61.433)
*Lepechinia schiedeana*	LBbgW01E	39.81 (26.278, 60.311)
*Montanoa ovalifolia*	ATbyW02L	48.75 (32.181, 73.858)
*Achryocline olata*	ATnaW02B	53.46 (35.286, 80.984)
*Lippia origanoides*	VEsaWCR-01	53.33 (35.205, 80.798)
*Hyptis mutabilis*	LMmeW02H	60.53 (39.958, 91.706)

Values in brackets are 95% confidence intervals (CI).

A repellency assay was then performed on the essential oils using *S.aegypti* mosquitoes and a modified version of the WHOPES arm-in-cage assay. The repellency EC_50_ values estimated for the oils with corresponding 95% confidence intervals were obtained from the regression of the proportion of repelled mosquitoes on the log-dose variable, using parallel logistic curves (Table 3). The fitted model for each oil, the observed data, the 95% confidence interval around the fitted model and the estimated EC_50_ are shown in Figures S1–12 in Supplementary Figures S1. It was found that the greatest repellency was also achieved by plants in the Verbenaceae family, reporting the lowest EC_50_ (Table 3). Once again the exception was found to be *L. origanoides* VEsaWCR-01. Although a version of the WHOPES recommended protocol was followed, where the same mosquitoes are used in multiple tests of different concentrations, a “carry-over” effect was observed particularly for *L. origanoides* VEbyW06B (*P*<0.01) and *L. origanoides* VEnaW02B (*P*<0.001) at the two greatest concentrations (1% and 10% (0.57–0.73 mg/cm^2^ on the skin). At these doses, the mosquitoes were less likely to bite, even in subsequent control experiments where no active ingredient was present on the volunteer’s arm suggesting that the essential oils had a residual negative effect on mosquito biting behaviour. For these reasons the repellency experiments with the two greatest doses for *L. origanoides* VEbyW06B and *L. origanoides* VEnaW02B were repeated using fresh mosquitoes to give a “true” repellency avoiding the complication of the “carry-over” effect and giving a more accurate EC_50_. However, this “carry over” or residual effect is in itself interesting and may suggest that the essential oils have a residual effect on the olfactory system.

**Table 3:** Estimated EC_50_ (mg/cm^2^) of repellency with 95% confidence interval (CI) for the 13 essential oils.

Sample	Code	EC_50_(95% CI)
*Lippia origanoides*	VEbyW06B	0.50 (0.31, 0.79)
*Ocinum campechianum*	LMsuW02B	0.51 (0.32, 0.81)
*Lippia alba*	VEboW02E	0.58 (0.37, 0.90)
*Lepechinia schiedeana*	LBbgW01E	0.60 (0.39, 0.94)
*Lippia origanoides*	VEnaW02B	0.61 (0.39, 0.95)
*Lippia alba*	VEbgW01E	0.62 (0.40, 0.97)
*Lippia origanoides (Thymol)*	VEsaWCR-02	0.79 (0.51, 1.23)
*Achryocline olata*	ATnaW02B	0.85 (0.55, 1.32)
*Lepechinia betonicifolia*	LTcuW24E	0.96 (0.63, 1.46)
*Lippia origanoides (Carvacrol)*	VEsaWCR-01	1.10 (0.69, 1.75)
*Condylidium cuatrecosasii*	ATsaW13B	1.11 (0.70, 1.78)
*Hyptis mutabilis*	LMmeW02H	1.13 (0.70, 1.81)
*Montanoa ovalifolia*	ATbyW02L	1.52 (0.89, 2.59)

**Figure 1:**
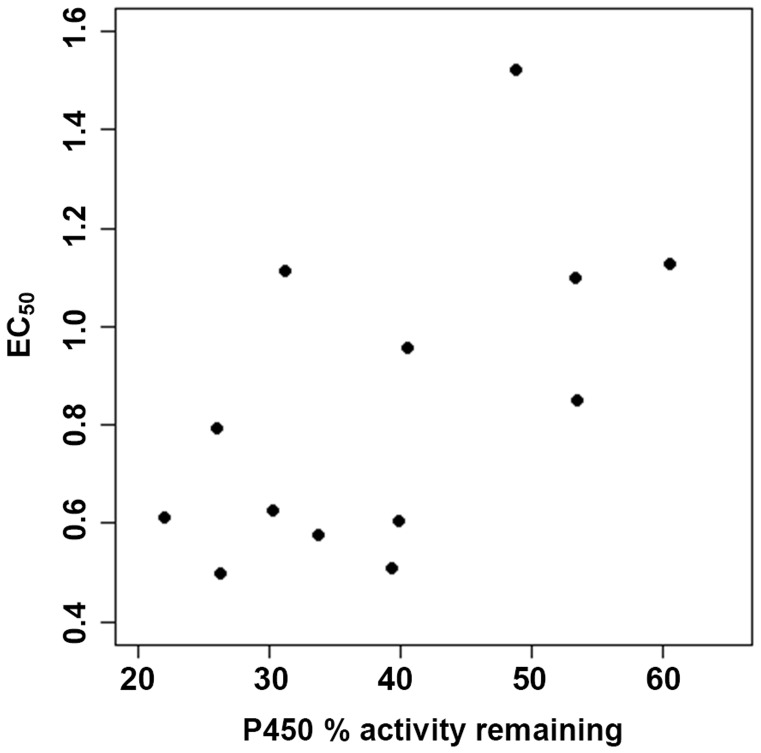
Estimated mean percentage activity remaining of P450 enzymes plotted against EC_50_ (mg/cm^2^) values of repellency. The agreement between P450 inhibition and repellency is evident; an increase in P450 inhibition is clearly linked to an increase in repellency. Plotted values as in Tables 2 and 3.

**Figure 2:**
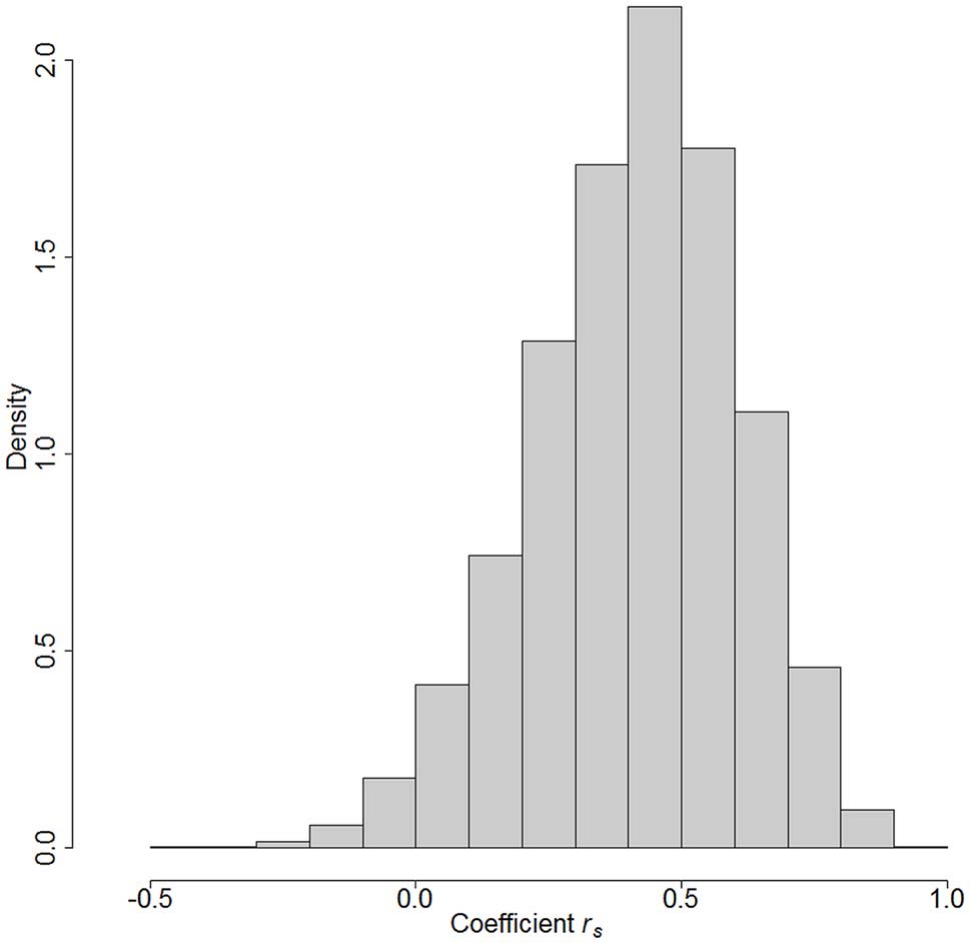
Bootstrap distribution of 10,000 correlation coefficients obtained as described in the Materials and Methods section in the text. The exercise of determining the degree of agreement (i.e. correlation) between the P450 and the EC_50_ ranking systems was replicated 10,000 times to measure the variation in correlation estimates. The figure shows how the vast majority of the replicate correlation coefficients are positive, and it enables visualisation of both the value upon which the correlation coefficients are centred and the extent of their spread.

Rank tests between the P450-inhibition and repellence activity data demonstrated that there was a significant positive correlation between P450-inhibition and repellent activity of the oils (*P*  = 0.0249). This strong correlation can be clearly observed in Fig. 1; EOs with a high mean percentage activity remaining of P450 activity tend to also have a high EC_50_. Conversely, EOs with a low P450 activity remaining display a low EC_50_. After applying a parametric bootstrap procedure with 10,000 replications, the mean correlation coefficient (± SE) was calculated as 0.4127 (±0.1933) (Figure 2). Our experiments, therefore, suggest that an increase in the P450 inhibitory potency of essential oils is associated with an increase in repellency. That such a correlation was derived from looking at inhibition of P450 activity from a sample of rabbit liver may seem unlikely, but it should be noted that all P450 enzymes have a common motif surrounding the omnipresent haem moiety, thus although the enzymes’ substrate specificity may be either broad or specific, P450 inhibitors often act universally. An even closer correlation between P450 inhibition and repellency could be expected if the P450 enzymes from mosquitoes, and perhaps those associated with the olfactory system, were used as a source of activity. Nevertheless, the correlation is compelling, and it is of note that the two EOs that resulted in a carry-over effect were the two most potent P450 inhibitors.

Following GC-MS analysis, two of the major compounds in the most effective sample (VEbyW06B - *L. origanoides*) were identified as thymol and carvacrol. These two monoterpenes have already been shown to have repellent activity against mosquitoes [23], [24]. Thymol was found to represent 50.1% of the sample, whilst carvacrol made up 14% of the sample. Similarly, in a different chemotype from *L. origanoides* (VEnaW02B), which also demonstrated good repellency, thymol was found to represent 71% of the sample and carvacrol 0.31%. Repellency tests were also done for carvacrol and thymol. The EC_50_ obtained for carvacrol was 0.39, almost as potent as DEET (0.31). Thymol (EC_50_ 0.87) was less potent. However, repellency may depend upon ratios of repellent chemicals and perhaps lack of repellence antagonists rather than simply the concentration of carvacrol present. The pure compounds were also tested for P450 inhibition (3 µL of 10% solution)), although these cannot be compared directly to the P450 inhibition results for the EOs. Carvacrol inhibited P450 activity 84.6% and thymol 86.9%. Thus, both were P450 inhibitors, with no significant potency differences between them.

To evaluate which compounds in an essential oil are detected by the receptors in the antennae of the mosquitoes, electroantennogram (EAG) and coupled gas chromatography-electronantennography (GC-EAG) analyses were performed with two different samples. One highly repellent (*L. origanoides* VEbyW06B) and one ineffective (*Lepechinia betonicifolia* LTcuW24E) extract were chosen for the analysis. There was a significantly greater EAG response to the ineffective repellent (sample LTcuW24E - *L. betonicifolia*) than to the effective one (*L. origanoides*) (t_10_ = 2.53; p = 0.029). This lack of response to the effective repellent may suggest that the repellent activity of the EO does not function through detection by the olfactory receptors on the antennae. Indeed, during GC-EAG analysis neither thymol nor carvacrol was detected by the receptors located in the antennae of *S. aegypti*, despite them having been shown to have repellent properties. There were 15 other peaks from *L. origanoides* (VEbyW06B) associated with EAG activity which may or may not play a role in the repellent activity of the EOs. These compounds still need to be identified by gas chromatography-mass spectrometry (GC-MS).

If P450 inhibition is to be considered an aspect of repellency, however, it must be considered how such a mechanism is viable. Some repellents appear to be capable of repellence or attraction according to concentration [25]. It has been proposed [26] that when an odour combines with a specific olfactory receptor to form a complex, there is an immediate ionotropic pathway and a slower metabotropic pathway initiated. It is possible that the metabotropic pathway depends upon the action of P450s at some point during the cascade. In vertebrates, the sustentacular cells provide a rich source of P450 enzymes, possibly for this purpose [27]. If this were the case, one might expect an attraction from the complex formation via the ionotropic pathway, but inhibition of attraction following inhibition of P450s along the metabotropic pathway. Similarly, in the presence of attractants (e.g. mosquitoes locating host skin) the metabotropic pathway would be broken with inhibition of P450s.

Another key component of the insect olfactory system is the production of odour degrading enzymes (ODEs) which break down odour ligands after they have bound to the olfactory receptor (OR). This process is vital to prevent the OR from continuing to respond which would cause confusion in the interpretation of olfactory signals. ODEs can be extracellular (present in the sensillum lymph) or intracellular (such as glutathione-*S*-transferase or cytochrome P450). The latter type are supposed to degrade odours entering the support cells of the sensilla [28], [29], [30], [31]. If the repellent compounds act by inhibiting the activity or production of those enzymes, as suggested by our experiments, an ORN for an attractant would continue to fire upon detection of olfactory stimuli as there are no enzymes to break them down. This could “confuse” or disorientate the insect and render it unable to successfully locate a host.

A further possibility is that the cocktail of compounds within the EOs contain a repellent separate from the P450 inhibitor, but the latter protects the former from metabolism within the mosquito body. The correlation then observed would be the result of greater protection of the repellent rather than P450 inhibition being the causative agent. This possibility would not hold for the observed effects with individual compounds in the pilot study, however.

Although we have not fully elucidated the mode of action relating to the association between P450 activity and repellency, we have demonstrated a significant correlation. As well as this indicating a new mechanism for repellency, with further investigation and refinement, a high throughput method of identifying novel repellent active ingredients using rapid biochemical screening assays based on the detection of P450 inhibition could be developed.

## Materials and Methods

### Essential Oils

Thirteen essential oil samples extracted from Colombian plants were provided by CENIVAM (Research Center of Excellence, Industrial University of Santander, Bucaramanga, Colombia) and evaluated as repellents against *S.aegypti* females. All necessary permits were obtained for the described field studies from Colombian Ministry of Environment, Housing and Territorial Development. The plants, which belong to four different families, were collected from their natural habitats in different parts of Colombia. The collection, identification and essential oil extraction were carried out by CENIVAM (Table 1). Essential oils were extracted by a microwave radiation-assisted hydrodistillation technique [32] using 400 g of fresh stems and leaves.

### Insects

*Stegomyia aegypti* LSHTM strain (‘refm’ strain obtained from the London School of Hygiene and Tropical Medicine) used in this study were reared in 30×30×30 cm cages in rooms maintained at 27°C, 60–80% relative humidity, and a 12:12 light:dark cycle. Larvae were reared on guinea pig food pellets, and adults were fed on 10% sucrose solution. Females were fed with horse blood by using a Hemotek® system. The mosquitoes used in repellency tests were females, 5–10 days old, not blood-fed.

### Rabbit Liver Homogenisation

Approximately 10 mg fresh rabbit liver was homogenised on ice in 250 µL homogenisation buffer (0.1 M phosphate buffer, pH 7.6, containing 1 mM EDTA, 1 mM DTT, 1 mM PTU, 1 mM PMSF and 1.46 M sucrose) and then diluted with the same buffer to give a final of volume 1 mL. This was centrifuged at 10 000×g for 10 min and the supernatant taken as the source for oxidase (P450) activity.

### Repellency Test

To estimate the effective dose of each essential oil, repellency tests were carried out following procedures recommended by the World Health Organization Pesticides Evaluation Scheme (WHOPES) [33], with some modifications. In addition, the standard repellent, DEET, was evaluated in the same way. A single volunteer was used for all the assays.

Each essential oil was serial diluted (0.01%, 0.1%, 1% and 10%) in ethanol and applied to the forearm of the volunteer (344 cm^2^). The hand was covered with two nitrile gloves. Ethanol (250 µl) served as a negative control and DEET (10%) as a positive control.

Before and after each assay the test area was washed with simple soap and rinsed with water, then rinsed with a solution of 70% ethanol and dried with a paper towel. The use of fragrances, creams and other products were avoided for at least 12 hours prior the assays. The tests were carried out in an experimental room maintained at 26°C ±2°C.

Between 30–40 host-seeking mosquitoes were placed in a 30×30×30 cm cage with clear plastic sides (adapted from Megaview® Bugdorm 1) [5]. The untreated “control” arm (with 250 µl of ethanol) was inserted into the cage and the number of probing mosquitoes were recorded over 1 min. Then, the same arm was treated with the lowest dose of the essential oil and placed in the cage for another minute. This procedure was repeated for each additional incremental repellent dose. In the middle and at the end of the dose-response experiment, ethanol was again applied to the opposite untreated forearm as negative control. At the end of the test a positive control (250 µl 10% DEET) was applied. Three replicates per EO were assayed using different batches of mosquitoes over several days. The (negative) control arm per assay was randomized to compensate for effects of using different arms. Replicate assays were randomly allocated to either morning or afternoon sessions to ensure that no effects were introduced by time of day.

### Determination of P450 Inhibition

P450 activity (from a microsomal preparation of rabbit liver) was measured *in vitro* according to Ullrich and Weber [34] and adapted to the microplate format as described by De Sousa *et al*. [35]. Briefly, 7-ethoxycoumarin was dissolved in ethanol to make a 20 mM stock solution and diluted by the addition of 0.1 M sodium phosphate buffer (pH 7.8) to give 0.5 mM. Dilute enzyme (47 µL) was added to each well, followed by the addition of 3 µL ethanol. After 10 mins, 80 µL ethoxycoumarin was added. The microplate was incubated for 5 min at 30°C and the reaction was initiated by the addition of 10 µL of 9.6 mM NADPH. Activity was read in a Spectramax Gemini XPS (Molecular Devices, Menlo Park CA) for 60 min, with readings taken every 5 min, using an excitation wavelength 370 nm and an emission wavelength of 460 nm. To measure inhibition, stock solutions (1%) of geranyl acetone, 6-methyl-heptene-2-one, 1-octen-3-ol or (10%) of the essential oils in ethanol were prepared. Dilute enzyme (47 µl) was incubated with 3 µl inhibitor for 10 mins, after which 80 µL ethoxycoumarin was added and the assay completed as before. Three replicates of each essential oil were assayed.

### Analysis of Volatile Organic Compounds

The essential oils were analysed for volatile organic compounds by high resolution gas chromatography (GC) on a non-polar capillary column (30 m×0.32 mm inner diameter×0.25 µm film thickness), (HP-5, J & W Scientific) using a HP6890N GC (Agilent Technologies, UK) fitted with a cool-on-column injector, a deactivated retention gap (1 m×0.53 mm inner diameter) and a flame ionisation detector (FID). The GC oven temperature was maintained at 30°C for 1 min after sample injection and then raised by 5°C min^−1^ to 150°C, then 10°C min^−1^ to 250°C. The carrier gas was hydrogen.

Coupled gas chromatography-mass spectrometry (GC-MS) analysis was performed on a HP 5972 MSD (Mass Selective Detector) and a HP 5890 GC fitted with a non-polar column (50 m×0.32 mm inner diameter×0.5 µm film thickness), (HP-1J & W Scientific) and a cool on-column injector (Gerstel TDS3). The GC oven temperature was maintained at 40°C for 5 min and then programmed at 5°C min^−1^ to 250°C. Ionisation was by electron impact at 70 eV, 250°C (source temperature).

Tentative identifications were confirmed via peak enhancement by co-injection with authenticated standards on both polar (DB-wax, 30 m×0.32 mm inner diameter×0.5 µm film thickness, J & W Scientific) and non-polar (HP-1, 50 m×0.32 mm inner diameter×0.5 µm film thickness, J & W Scientific) capillary columns using a HP6890 GC (Agilent Technologies, UK) fitted with a cool-on-column injector, a deactivated retention gap (1 m×0.53 mm inner diameter) and a flame ionisation detector (FID). The GC oven temperature was maintained at 30°C for 1 min after sample injection and then raised by 5°C min-1 to 150°C, then 10°C min-1 to 230°C. The carrier gas was hydrogen.

### Electroantennography (EAG) and Coupled Gas Chromatography-Electroantennography (GC-EAG)

Following the protocols described by Logan *et al* (2008), an EAG to establish the activity of EOs at increasing concentrations (0.01%, 0.1% and 1%) and GC-EAG to locate peaks within mixtures that could be detected by the antennae of mosquitoes were performed. Adult females *S. aegypti* were cooled on ice for 30 sec before removing the head and thence tips of both antennae. The indifferent electrode was inserted into the back of the head, and the distal ends of both antennae were inserted into the recording electrode. Recordings were made with Ag/AgCl electrodes inserted into glass pipettes filled with a saline solution (insect ringer–7.55 g NaCl, 0.64 g KCl, 0.22 g CaCl_2_, 1.73 g MgCl_2_, 0.86 g Na_2_HCO_3_, and 0.61 g Na_3_PO_4_ L^−1^ water). Electrodes were connected to an Autospike interface box and an AC/DC amplifier UN-06 (Syntech). Preparations were held in a continuous, humidified, and charcoal-filtered air stream (1 Lmin^−1^, Syntech Stimulus Controller CS-02, Syntech) that came from a glass tube outlet that was positioned 0.5 cm from the preparation. A GC93A gas chromatograph was used to separate the components of mixtures on a HP-1 column. The oven temperature was maintained at 40°C for 2 min and then programmed at 5°C min^−1^ to 100°C and then at 10°C min^−1^ to 250°C. The carrier gas was hydrogen. As a reference stimulus, hexane was applied at the beginning, in the middle and at the end of the experiment. The EAG experiments were repeated six times, and the GC-EAD repeated three times for each EO evaluated.

## Statistical Analyses

### Analysis of Repellency Data

#### 1. Testing for a carry-over effect and, if found, repeating the problematic doses

When using certain EOs, it was observed that mosquitoes were less responsive as time went by, even when presented to the negative control. To test for the presence of this “carry-over” effect, the proportion of probing mosquitoes recorded for the negative control arm was statistically analysed. The negative control counts were extracted, converted into proportions and analysed for significant differences between the control count at the beginning and the control count at the end of an experiment. If evidence for significant differences was found, then it was concluded that the EO had a carry-over effect on mosquitoes. Assays for the EOs detected as problematic were then repeated, including only the two highest doses.

To conduct the analysis of carry-over effects, an ante-dependence model of first order [36] was fitted to the negative control proportions (transformed into 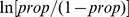]). The ante-dependence model accounted for the correlation structure that exists between assays that used the same batch of mosquitoes over time. The model was estimated using residual maximum likelihood (REML) methods [37] in GenStat for Windows [38]. The fixed component of the ante-dependence model was set to the product between *time* (indicating the time point at which the record was made; beginning, middle or end of experiment), and *oil* (indicating the EO applied). The random component associated the records taken on the same batch of mosquitoes over time. After finding statistical significance for the main effects of *time* and *oil,* pairwise comparisons between the estimated average response for a given *oil* at *time* “beginning” and at *time* “end” were conducted using a two-tailed *t* statistic.

#### 2. Adjusting with respect to the negative control

Once substituting the counts suspected to have a carry-over effect, the data were adjusted by the number of probing mosquitoes on the negative control arm. A repellency experiment consisted of records made at consecutive time points; from time point 1 to time point 8. Records at times 1, 4 and 7 referred to negative control counts whereas time points 2, 3, 5, 6 and 8 contained counts for four incremental EO doses plus the positive control. Note, therefore, that a variable on only time points 2, 3 5 and 6 is a surrogate for *dose*.

Let 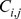 denote the observed negative control count at time point *i* (*i* = 1, 4, 7) for an experiment with EO *j* (j = 1,…,13).The *expected* negative control count at time points 2, 3, 5 and 6, for an experiment with EO *j,* was calculated as
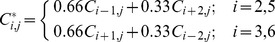



A control-adjusted proportion of repelled mosquitoes by EO *j* at time point *i* was computed as
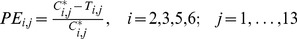
where 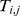 denotes the observed count of probing mosquitoes at time point *i* (*i* = 2, 3, 5, 6) when assaying EO *j* (*j* = 1, …, 13). Thus, doses with no repellence power scored 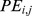 values close to zero, whereas strongly repellent doses achieved 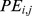
’s close to one.

#### 3. Fitting non-linear parallel curves

The interest was to model the relationship between the control-adjusted proportion of repelled mosquitoes by EO *j*, and the explanatory variable *dose*. A logistic curve
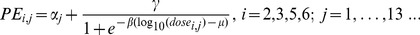
(1)was fitted to the control-adjusted proportion for the *j*th essential oil at dose *i*. Here, the dose-response relationship was assumed to follow equal parameters 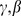 and 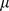 across all EOs, but an oil-specific origin 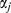. In practical terms, this means that the logistic curves for different oils were constrained to being parallel to one another, but free to having their own interception with the “*y*”-axis (note that, in our notation, the *y*-axis is actually the *PE*-axis).

Before fitting model (1) to the control-adjusted proportions, a test of parallelism was conducted to verify that parallel curves provided a reasonable fit to the data. The test was favourable and so the assumption of parallel curves was reasonable. Estimates of parameters in (1), together with their standard errors, were obtained using the Gauss-Newton method [39] of the FITNONLINEAR directive in GenStat for Windows [38]. All 13 logistic curves were simultaneously fitted.

#### 4. Estimating the EC_50_s

To estimate the dose that on average would produce 50% response (EC_50_), equation (1) was equalled to 0.5 and solved for the *dose* variable as
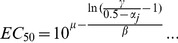
(2)


An EC_50_ for EO *j* was estimated by plugging into equation (2) the estimates for parameters 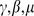 and 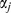 obtained as described in the previous section. The RFUNCTION directive in GenStat [38] was used for this calculation. The directive also generated a standard error for EC_50_ which was used, together with the 95% two-sided critical value of the Student’s t distribution with 143 degrees of freedom, to calculate a 95% confidence interval around the EC_50_ for EO *j*. Both the EC_50_ for the *j*th oil and its associated error were used in the rank tests described later in this paper.

### Analysis of P450 Inhibition

The percentage activity remaining of enzyme P450 measured on the *k^th^* replicate of EO*j,* denoted as *y_jk_* (*k = 1,2,3; j = 1,…,13*),was fitted using the linear model.
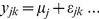
(3)where µ*_j_* represents the mean percentage enzyme activity remaining for EO*j,* and *ε_jk_* is the random variation, around *µ_j_*, for the *k^th^* replicate of EO*j.* The *ε_jk_* is assumed normally and independently distributed with zero mean and variance *σ^2^*. The parameters of interest in this analysis were the mean percentage enzyme activity*µ_j_*, and its associated standard error (SE*_j_*), 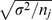, where *n_j_* is the replication number for EO *j*. Both quantities were used for the rank tests described below.

Model (3) was fitted to the log-transformed P450 data and parameter estimates were obtained using the ANOVA directive of the GenStat software package (Payne *et al*., 2010).

### Rank Tests between Repellency and P450 Inhibition

The mean percentage enzyme activity remaining (P450 analysis) and the EC_50_ (repellency analysis) estimated for the 13 oils were used to produce two rankings of oils; one according to the former quantity and a second one according to the latter. To determine the degree of agreement between the two ranking systems, the Spearman’s rank correlation coefficient, 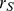, was calculated using the statistical software R [40]. A perfect agreement between the two systems exists if high ranks in the P450 analysis corresponded to high ranks in the repellence analysis (and vice versa).This would produce 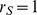.Perfect disagreement would be indicated by 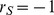.

The uncertainty in 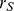 was approximated using a parametric bootstrap procedure. Briefly, it was assumed that the EC_50_ of EO *j* was normally distributed with mean at the estimated EC_50_ value and standard deviation as the corresponding EC_50_ estimated error. A ‘replicate’ set of 13 EC_50_ values was produced by sampling from the corresponding normal distribution, independently for each oil. Similarly, the distribution of mean percentage enzyme activity remaining of EO *j* was assumed normal with centre at the estimated *µ_j_* and standard deviation at SE*_j_*. A ‘replicate’ set of 13 mean percentage values was generated by sampling from the respective distributions. Once observing a ‘replicate’ dataset containing 13 repellence EC_50_s and 13 mean percentage activity values, a ‘replicate’ ranking of oils was produced together with its corresponding ‘replicate’ correlation coefficient. This replication exercise was repeated 10,000 times, resulting in 10,000 ‘replicate’ correlation coefficients. A histogram of these correlation coefficients was constructed to display the distribution of 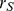.The proportion of sampled coefficients that were equal to, or less than, zero was used as an approximation to the *p*-value of a test ‘H_0_: no positive correlation between ranking systems’ versus ‘H_1_: positive correlation between ranks’.

## Supporting Information

Supplementary Figures S1 The fitted model for each oil, the observed data, the 95% confidence interval around the fitted model and the estimated EC_50_ are shown in Supplementary Figures; S1, ATbyW02L; S2, ATnaW02B; S3, ATsaW13B; S4, VEbgW01E; S5, LMmeW02H; S6, LTcuW24E; S7, VEbgW01E; S8, VEboW02E; S9, VEbyW06B; S10, VenaW02B; S11, VEsaWCR-01; S12, VEsaWCR-02.
(ZIP)
