# Supplementary figures and images for: Repellents Inhibit P450 Enzymes in Stegomyia (Aedes) aegypti
Source: PLoS One. 2012 Nov 13;7(11):e48698. doi: 10.1371/journal.pone.0048698 (PMC3496720; doi:10.1371/journal.pone.0048698)

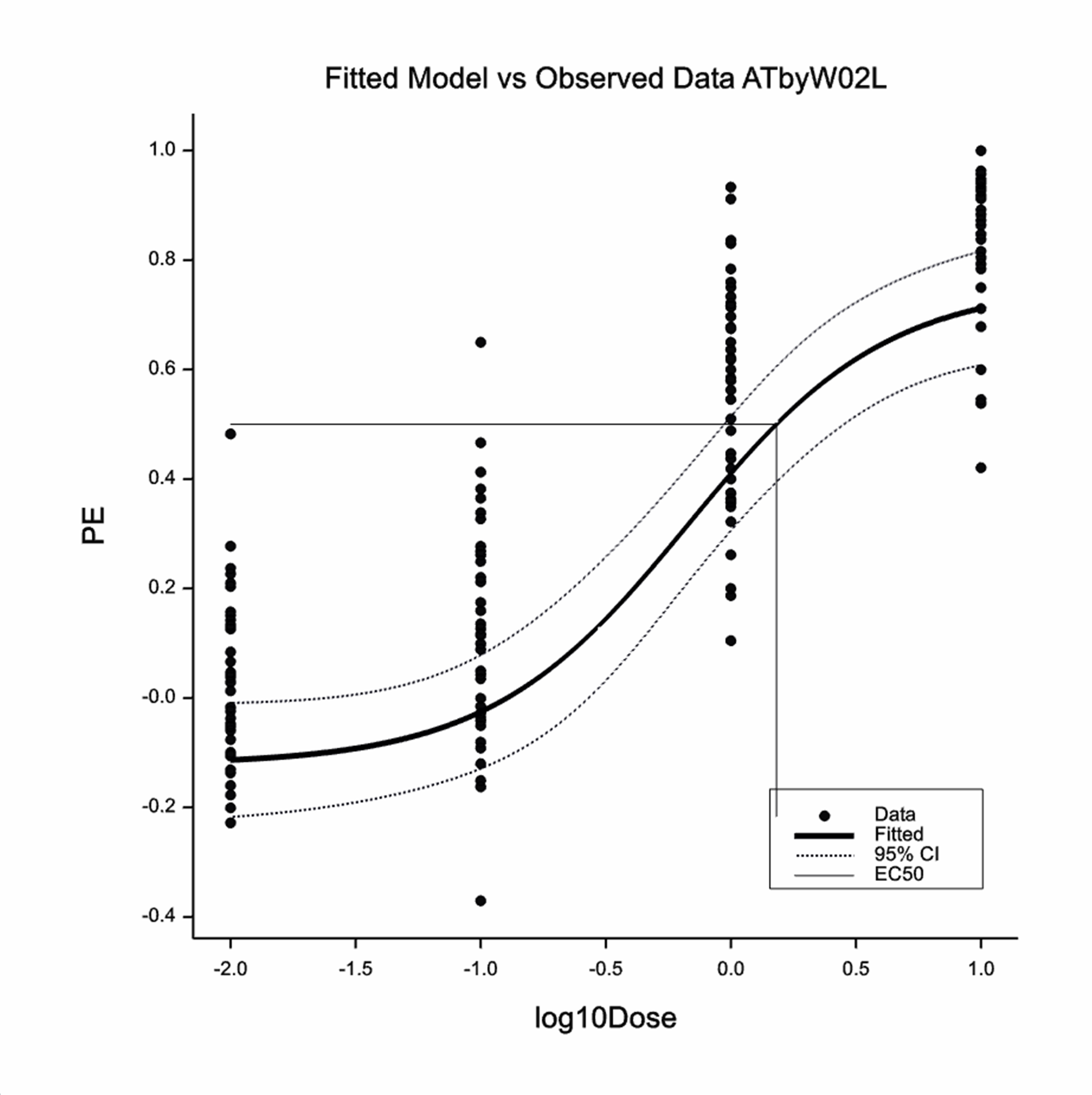

Supplement: Supplementary Figures S1 — The fitted model for each oil, the observed data, the 95% confidence interval around the fitted model and the estimated EC50 are shown in Supplementary Figures; S1, ATbyW02L; S2, ATnaW02B; S3, ATsaW13B; S4, VEbgW01E; S5, LMmeW02H; S6, LTcuW24E; S7, VEbgW01E; S8, VEboW02E; S9, VEbyW06B; S10, VenaW02B; S11, VEsaWCR-01; S12, VEsaWCR-02. (ZIP) [file pone.0048698.s001.zip › Fig S1.tif]

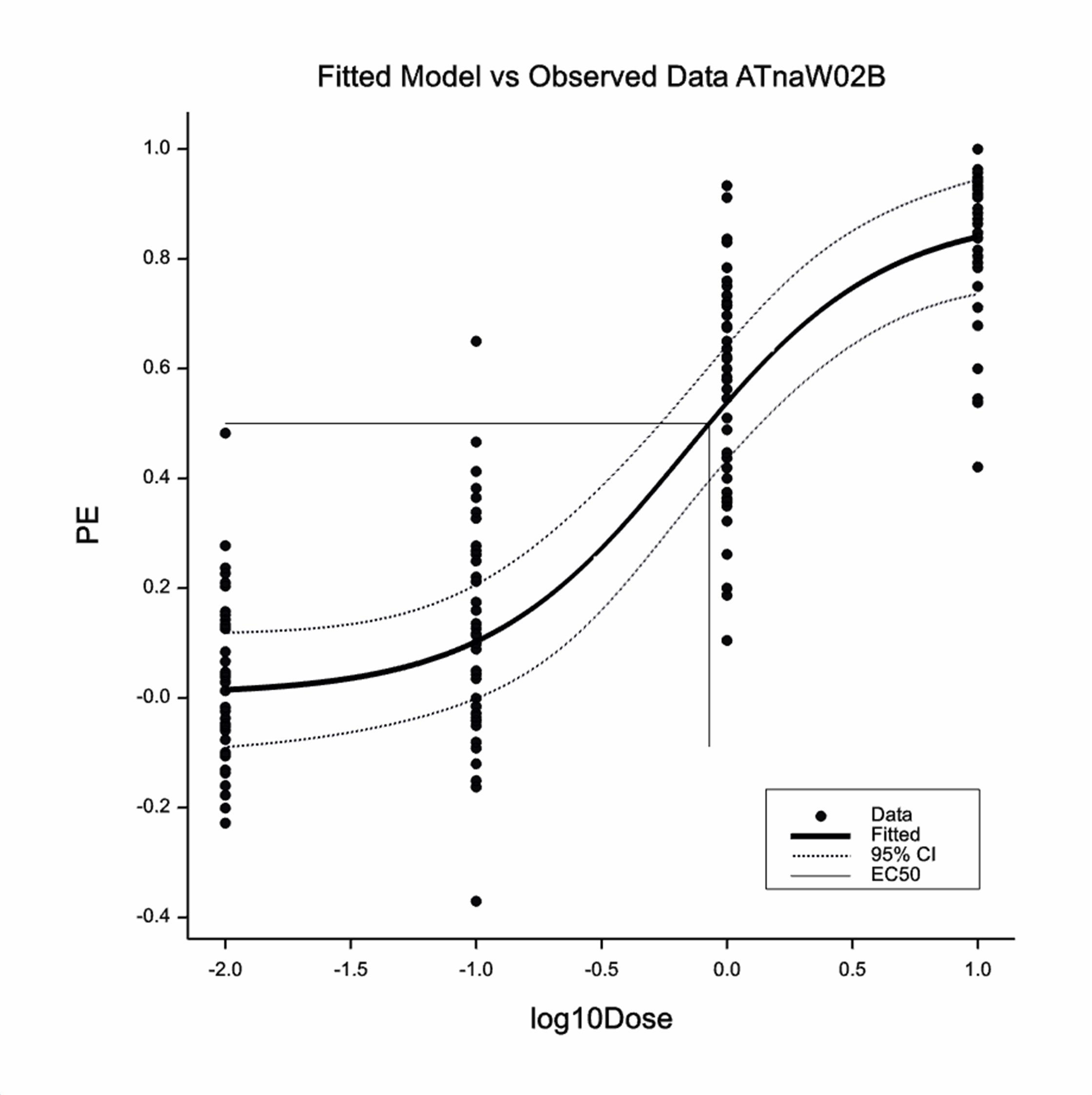

Supplement: Supplementary Figures S1 — The fitted model for each oil, the observed data, the 95% confidence interval around the fitted model and the estimated EC50 are shown in Supplementary Figures; S1, ATbyW02L; S2, ATnaW02B; S3, ATsaW13B; S4, VEbgW01E; S5, LMmeW02H; S6, LTcuW24E; S7, VEbgW01E; S8, VEboW02E; S9, VEbyW06B; S10, VenaW02B; S11, VEsaWCR-01; S12, VEsaWCR-02. (ZIP) [file pone.0048698.s001.zip › Fig S2.tif]

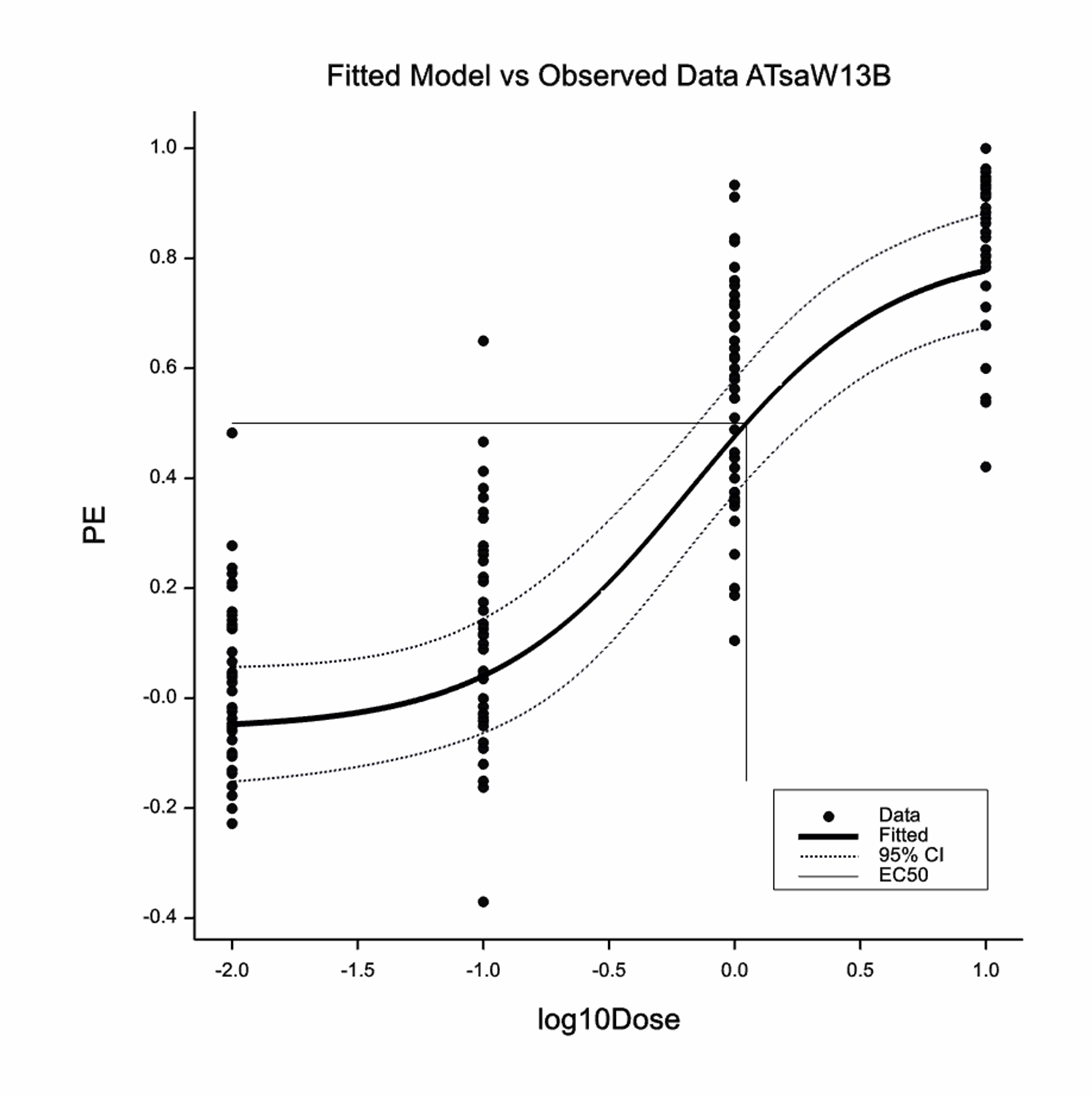

Supplement: Supplementary Figures S1 — The fitted model for each oil, the observed data, the 95% confidence interval around the fitted model and the estimated EC50 are shown in Supplementary Figures; S1, ATbyW02L; S2, ATnaW02B; S3, ATsaW13B; S4, VEbgW01E; S5, LMmeW02H; S6, LTcuW24E; S7, VEbgW01E; S8, VEboW02E; S9, VEbyW06B; S10, VenaW02B; S11, VEsaWCR-01; S12, VEsaWCR-02. (ZIP) [file pone.0048698.s001.zip › Fig S3.tif]

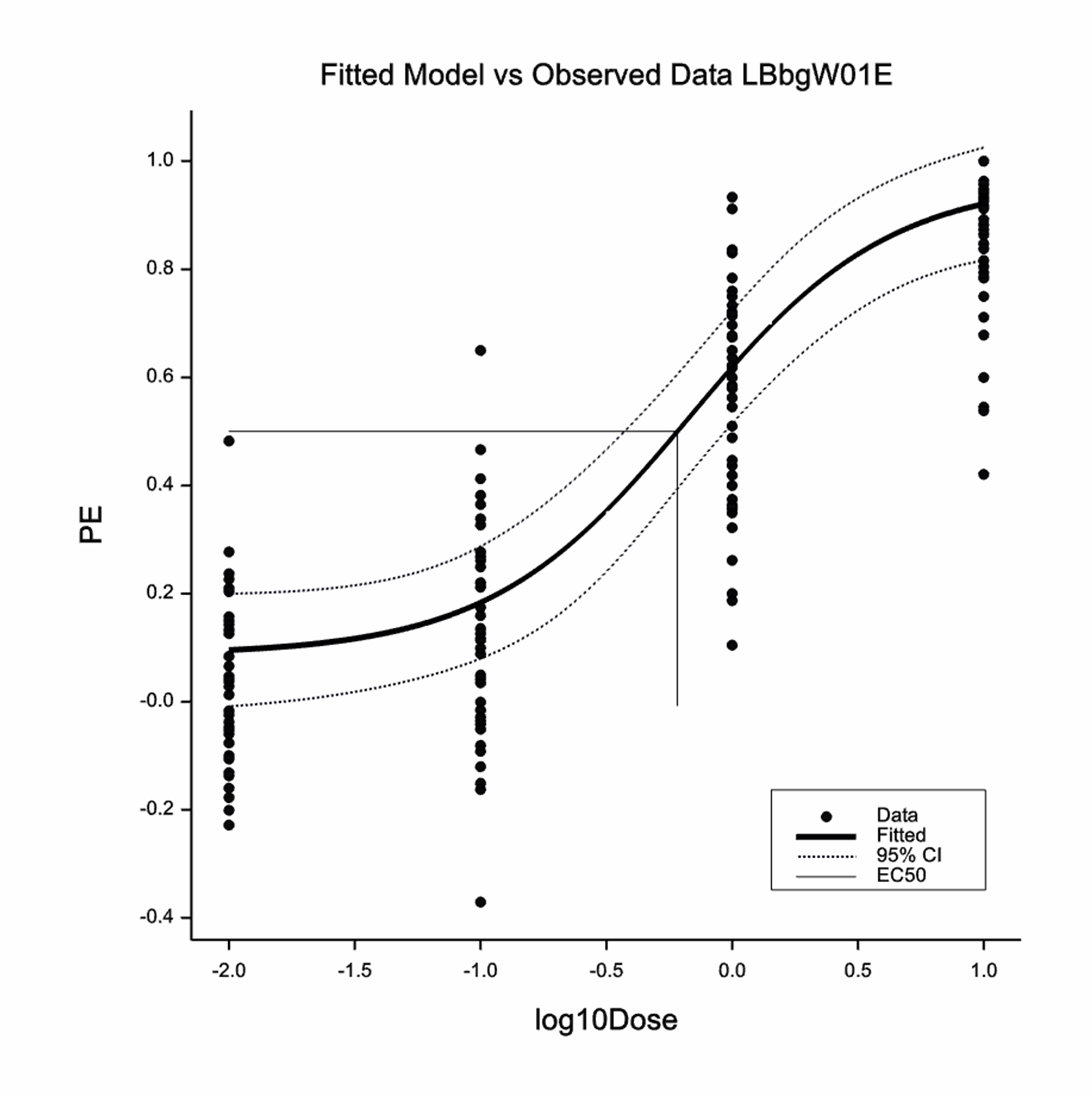

Supplement: Supplementary Figures S1 — The fitted model for each oil, the observed data, the 95% confidence interval around the fitted model and the estimated EC50 are shown in Supplementary Figures; S1, ATbyW02L; S2, ATnaW02B; S3, ATsaW13B; S4, VEbgW01E; S5, LMmeW02H; S6, LTcuW24E; S7, VEbgW01E; S8, VEboW02E; S9, VEbyW06B; S10, VenaW02B; S11, VEsaWCR-01; S12, VEsaWCR-02. (ZIP) [file pone.0048698.s001.zip › Fig S4.tif]

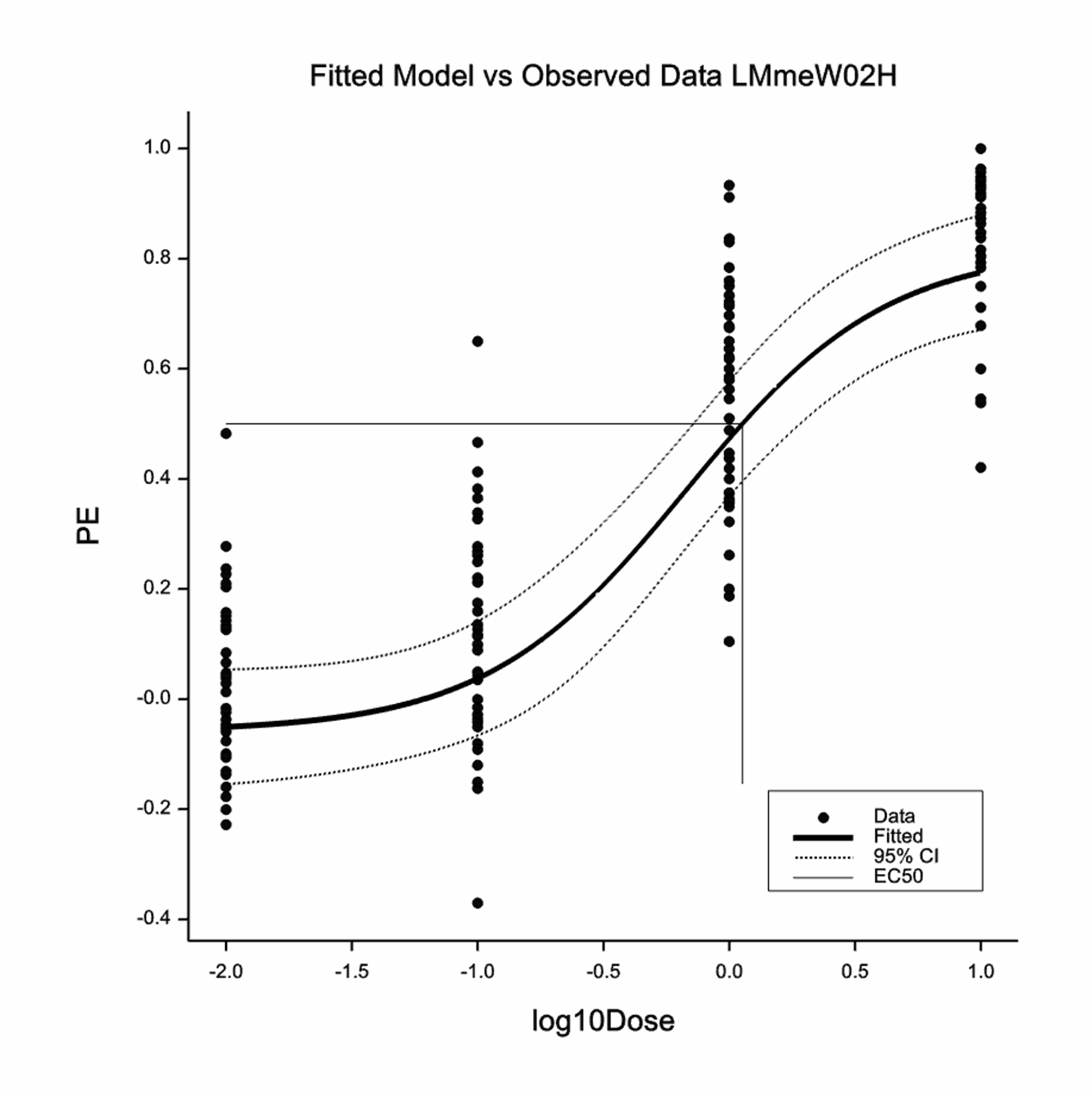

Supplement: Supplementary Figures S1 — The fitted model for each oil, the observed data, the 95% confidence interval around the fitted model and the estimated EC50 are shown in Supplementary Figures; S1, ATbyW02L; S2, ATnaW02B; S3, ATsaW13B; S4, VEbgW01E; S5, LMmeW02H; S6, LTcuW24E; S7, VEbgW01E; S8, VEboW02E; S9, VEbyW06B; S10, VenaW02B; S11, VEsaWCR-01; S12, VEsaWCR-02. (ZIP) [file pone.0048698.s001.zip › Fig S5.tif]

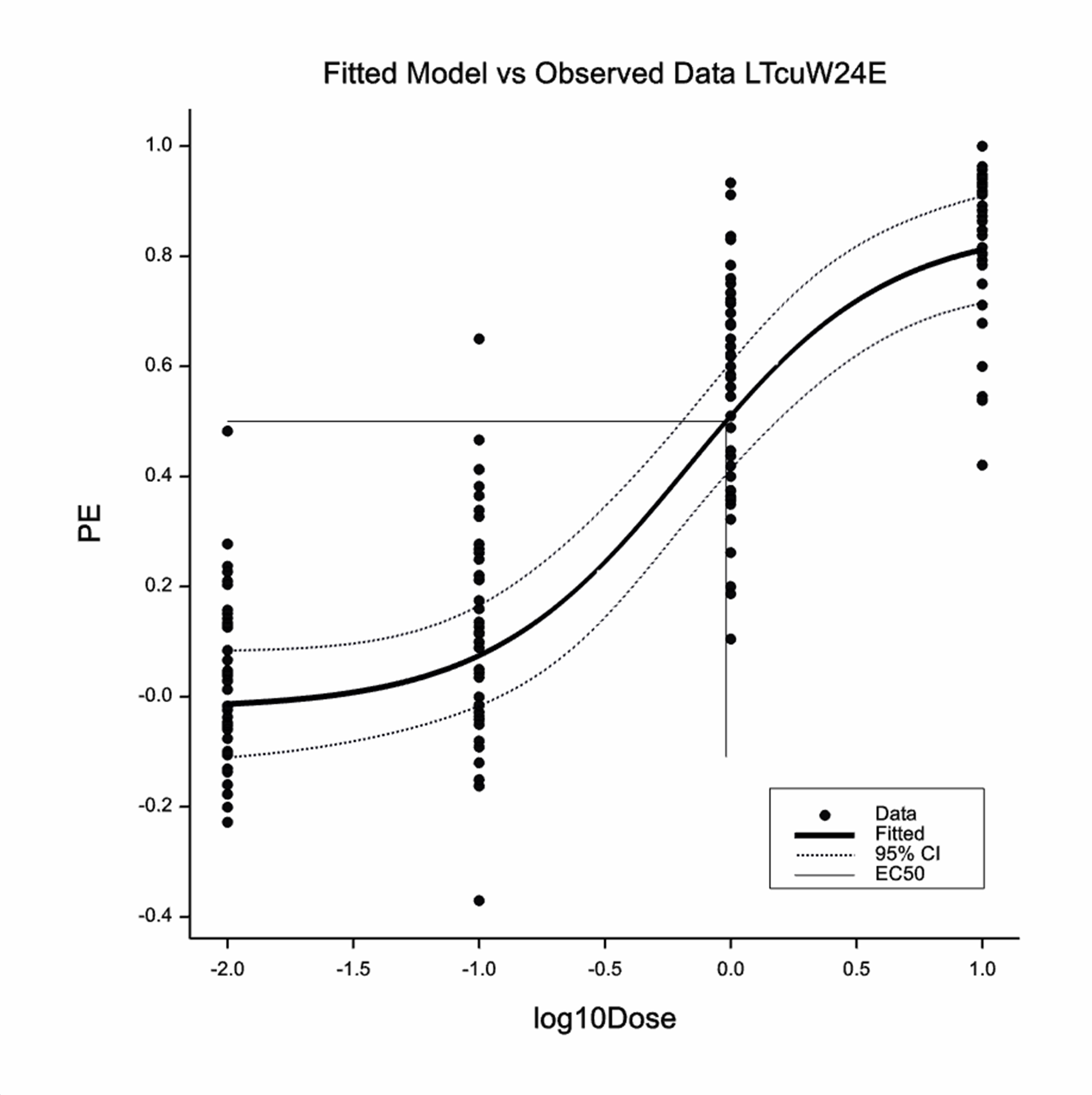

Supplement: Supplementary Figures S1 — The fitted model for each oil, the observed data, the 95% confidence interval around the fitted model and the estimated EC50 are shown in Supplementary Figures; S1, ATbyW02L; S2, ATnaW02B; S3, ATsaW13B; S4, VEbgW01E; S5, LMmeW02H; S6, LTcuW24E; S7, VEbgW01E; S8, VEboW02E; S9, VEbyW06B; S10, VenaW02B; S11, VEsaWCR-01; S12, VEsaWCR-02. (ZIP) [file pone.0048698.s001.zip › Fig S6.tif]

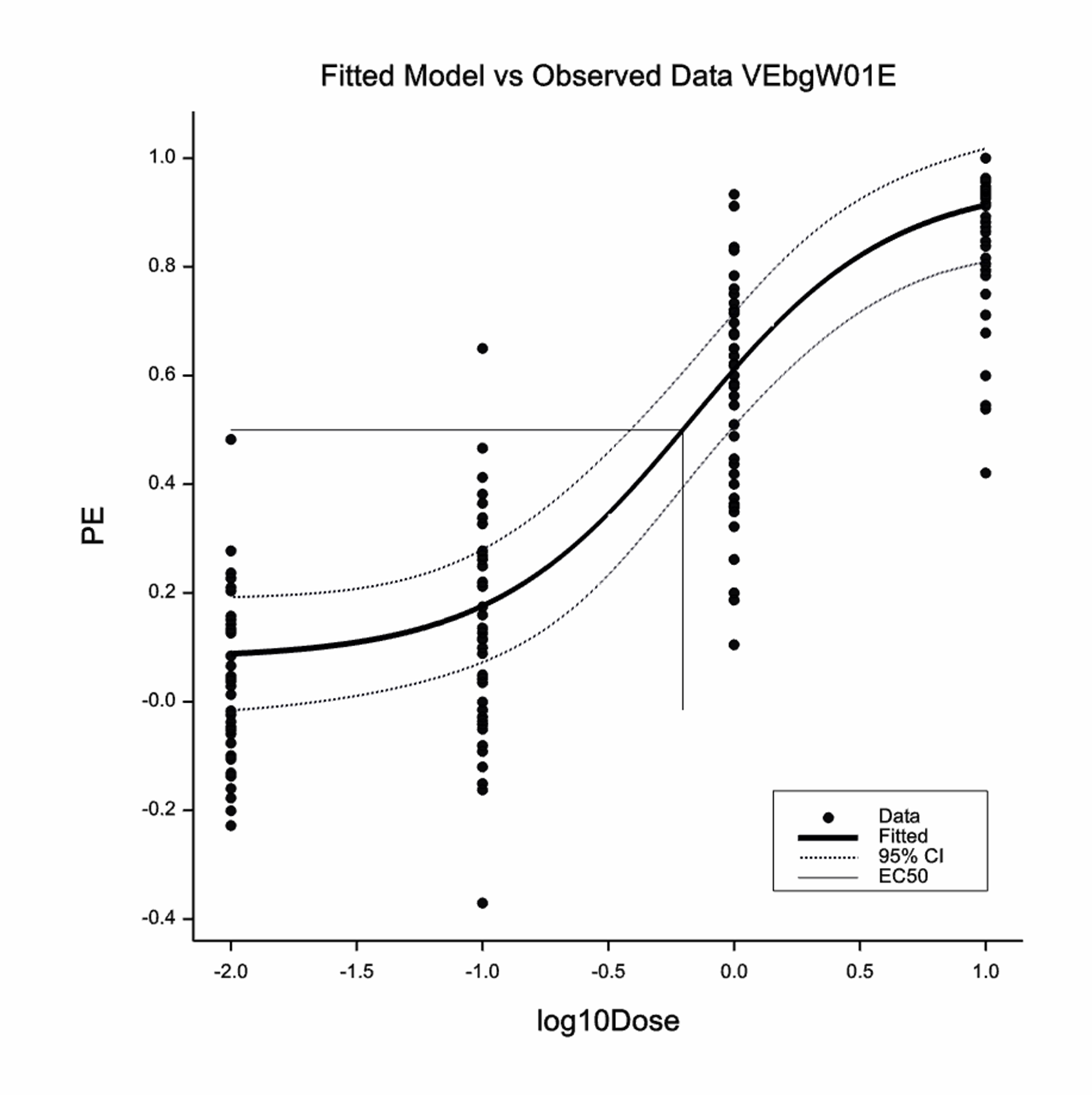

Supplement: Supplementary Figures S1 — The fitted model for each oil, the observed data, the 95% confidence interval around the fitted model and the estimated EC50 are shown in Supplementary Figures; S1, ATbyW02L; S2, ATnaW02B; S3, ATsaW13B; S4, VEbgW01E; S5, LMmeW02H; S6, LTcuW24E; S7, VEbgW01E; S8, VEboW02E; S9, VEbyW06B; S10, VenaW02B; S11, VEsaWCR-01; S12, VEsaWCR-02. (ZIP) [file pone.0048698.s001.zip › Fig S7.tif]

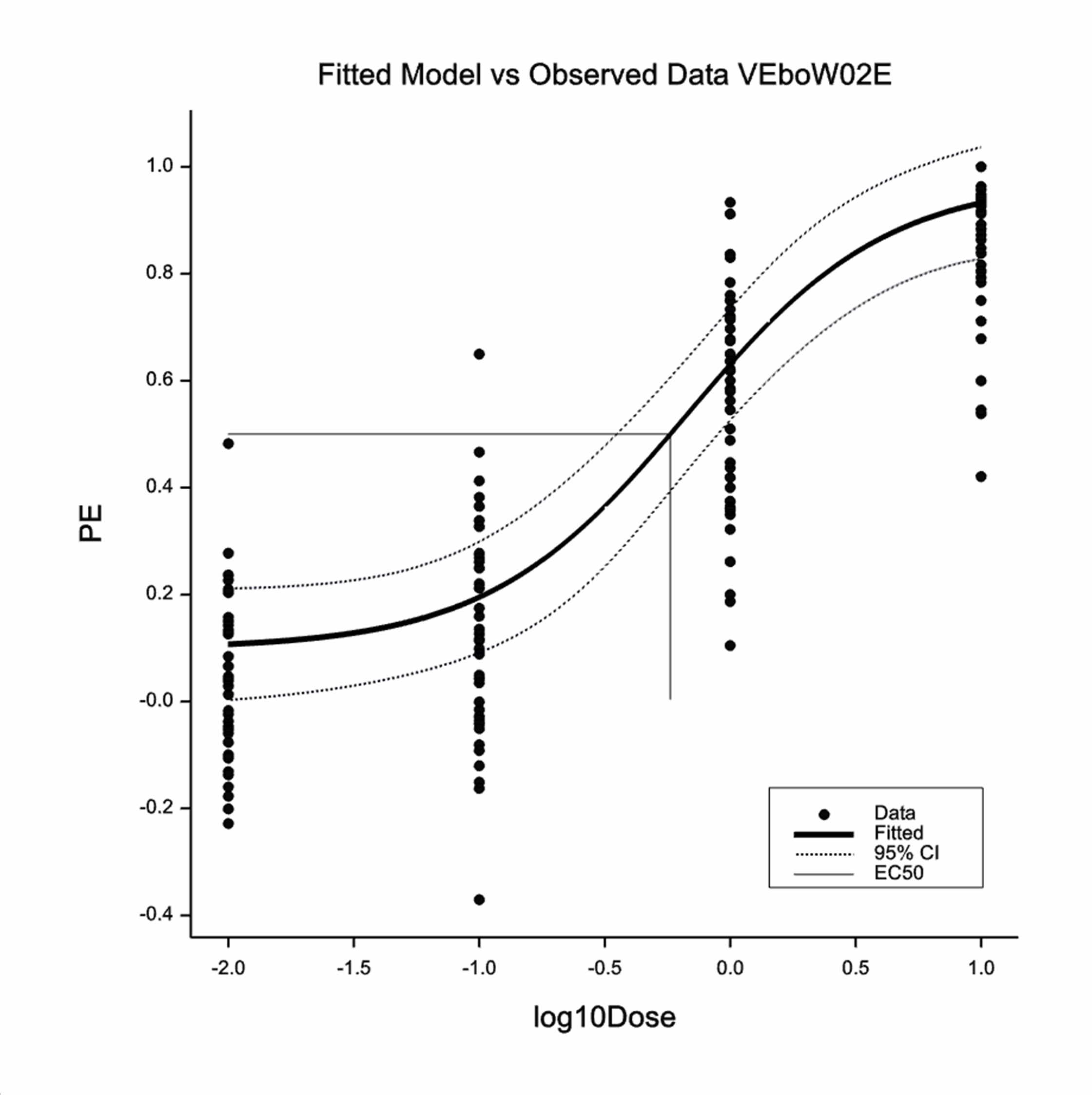

Supplement: Supplementary Figures S1 — The fitted model for each oil, the observed data, the 95% confidence interval around the fitted model and the estimated EC50 are shown in Supplementary Figures; S1, ATbyW02L; S2, ATnaW02B; S3, ATsaW13B; S4, VEbgW01E; S5, LMmeW02H; S6, LTcuW24E; S7, VEbgW01E; S8, VEboW02E; S9, VEbyW06B; S10, VenaW02B; S11, VEsaWCR-01; S12, VEsaWCR-02. (ZIP) [file pone.0048698.s001.zip › Fig S8.tif]

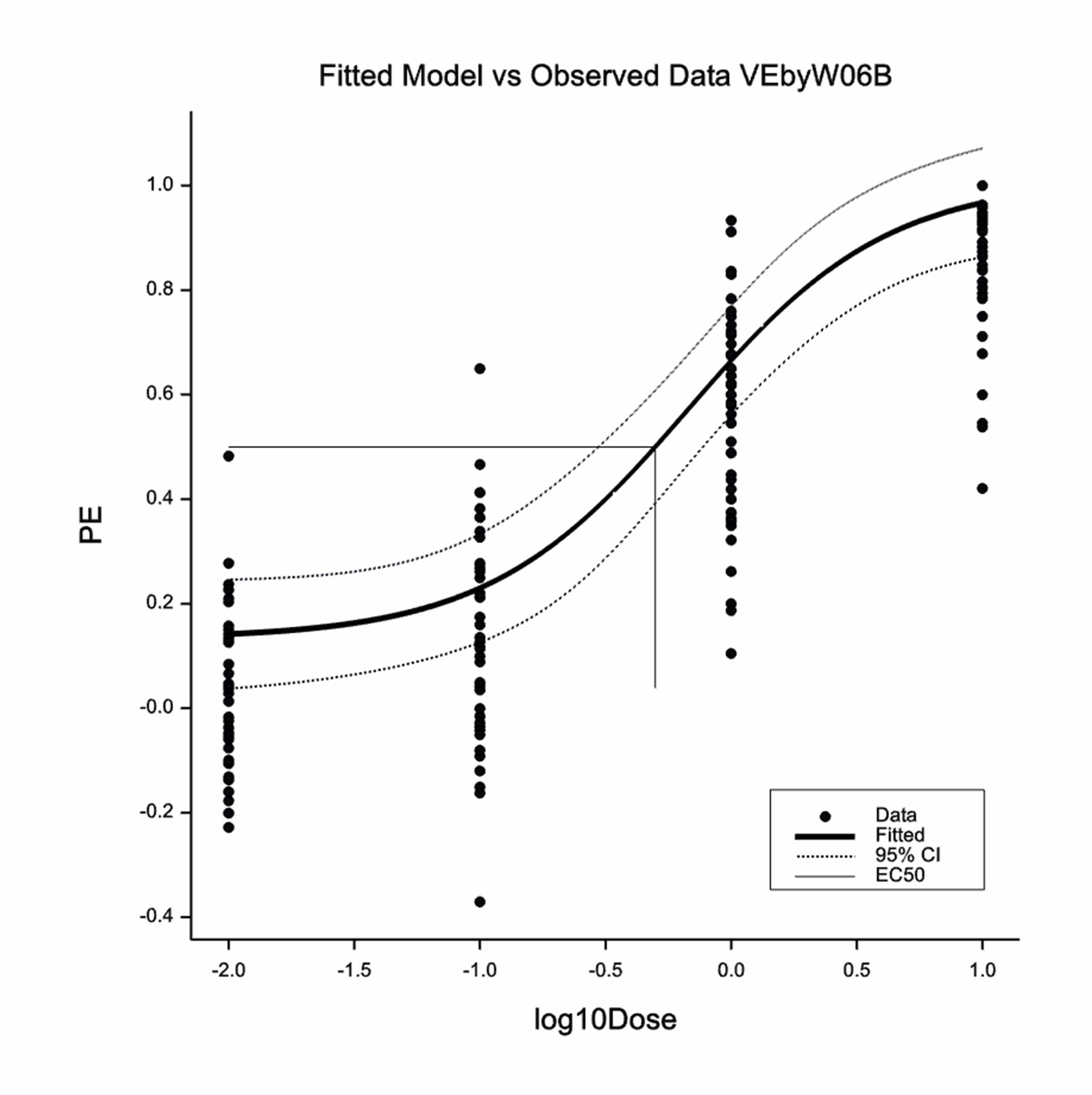

Supplement: Supplementary Figures S1 — The fitted model for each oil, the observed data, the 95% confidence interval around the fitted model and the estimated EC50 are shown in Supplementary Figures; S1, ATbyW02L; S2, ATnaW02B; S3, ATsaW13B; S4, VEbgW01E; S5, LMmeW02H; S6, LTcuW24E; S7, VEbgW01E; S8, VEboW02E; S9, VEbyW06B; S10, VenaW02B; S11, VEsaWCR-01; S12, VEsaWCR-02. (ZIP) [file pone.0048698.s001.zip › Fig S9.tif]

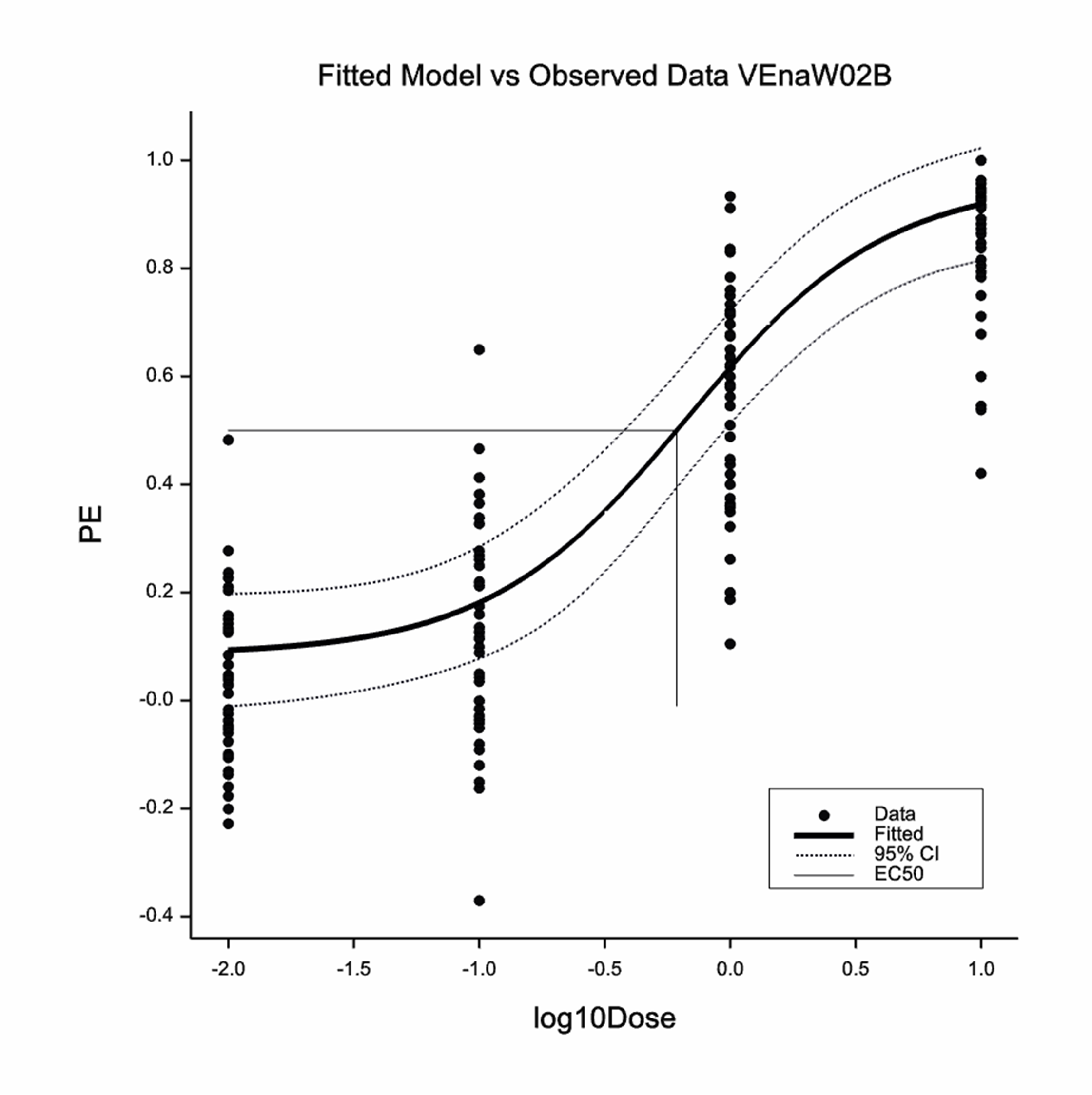

Supplement: Supplementary Figures S1 — The fitted model for each oil, the observed data, the 95% confidence interval around the fitted model and the estimated EC50 are shown in Supplementary Figures; S1, ATbyW02L; S2, ATnaW02B; S3, ATsaW13B; S4, VEbgW01E; S5, LMmeW02H; S6, LTcuW24E; S7, VEbgW01E; S8, VEboW02E; S9, VEbyW06B; S10, VenaW02B; S11, VEsaWCR-01; S12, VEsaWCR-02. (ZIP) [file pone.0048698.s001.zip › Fig S10.tif]

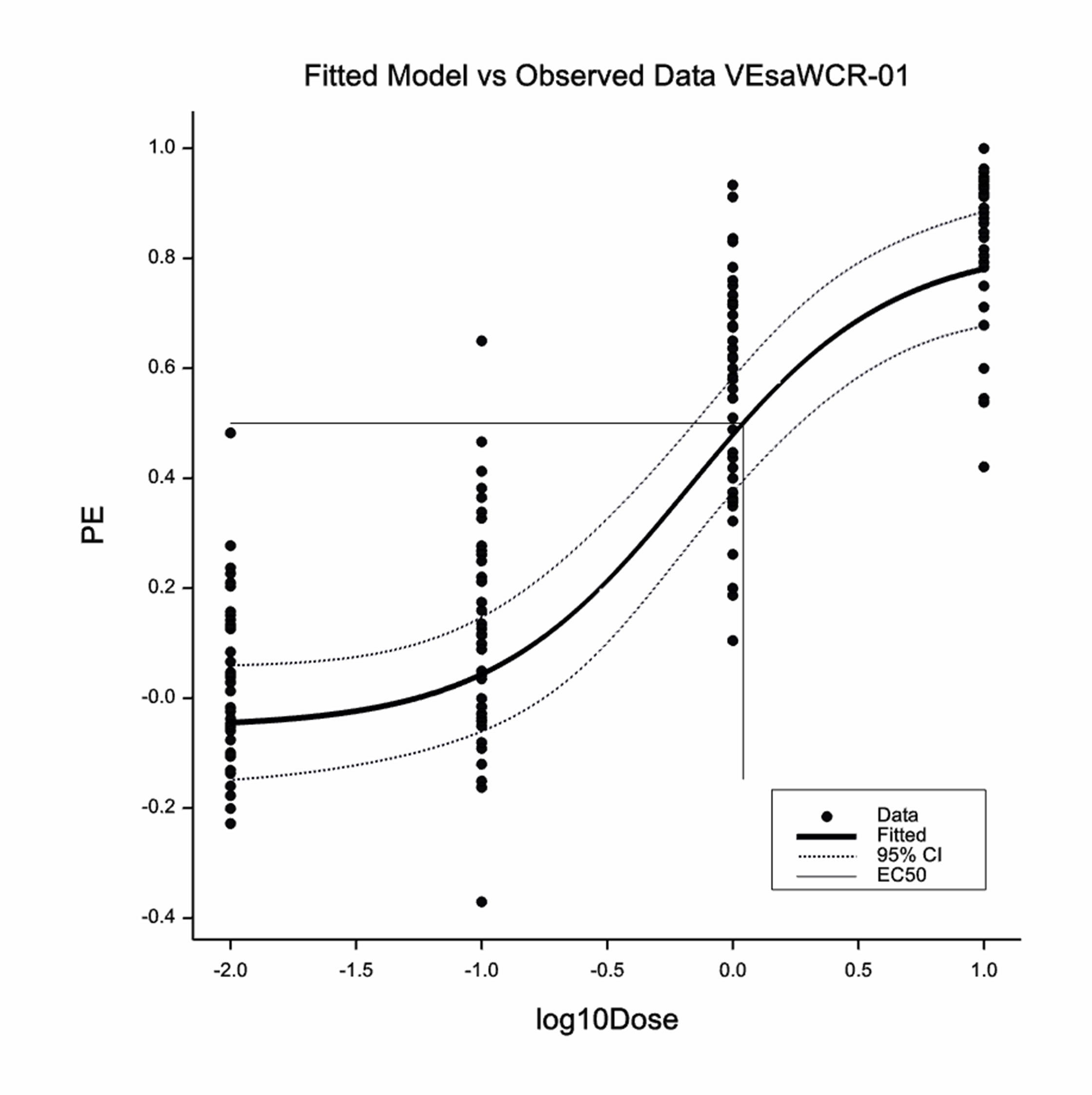

Supplement: Supplementary Figures S1 — The fitted model for each oil, the observed data, the 95% confidence interval around the fitted model and the estimated EC50 are shown in Supplementary Figures; S1, ATbyW02L; S2, ATnaW02B; S3, ATsaW13B; S4, VEbgW01E; S5, LMmeW02H; S6, LTcuW24E; S7, VEbgW01E; S8, VEboW02E; S9, VEbyW06B; S10, VenaW02B; S11, VEsaWCR-01; S12, VEsaWCR-02. (ZIP) [file pone.0048698.s001.zip › Fig S11.tif]

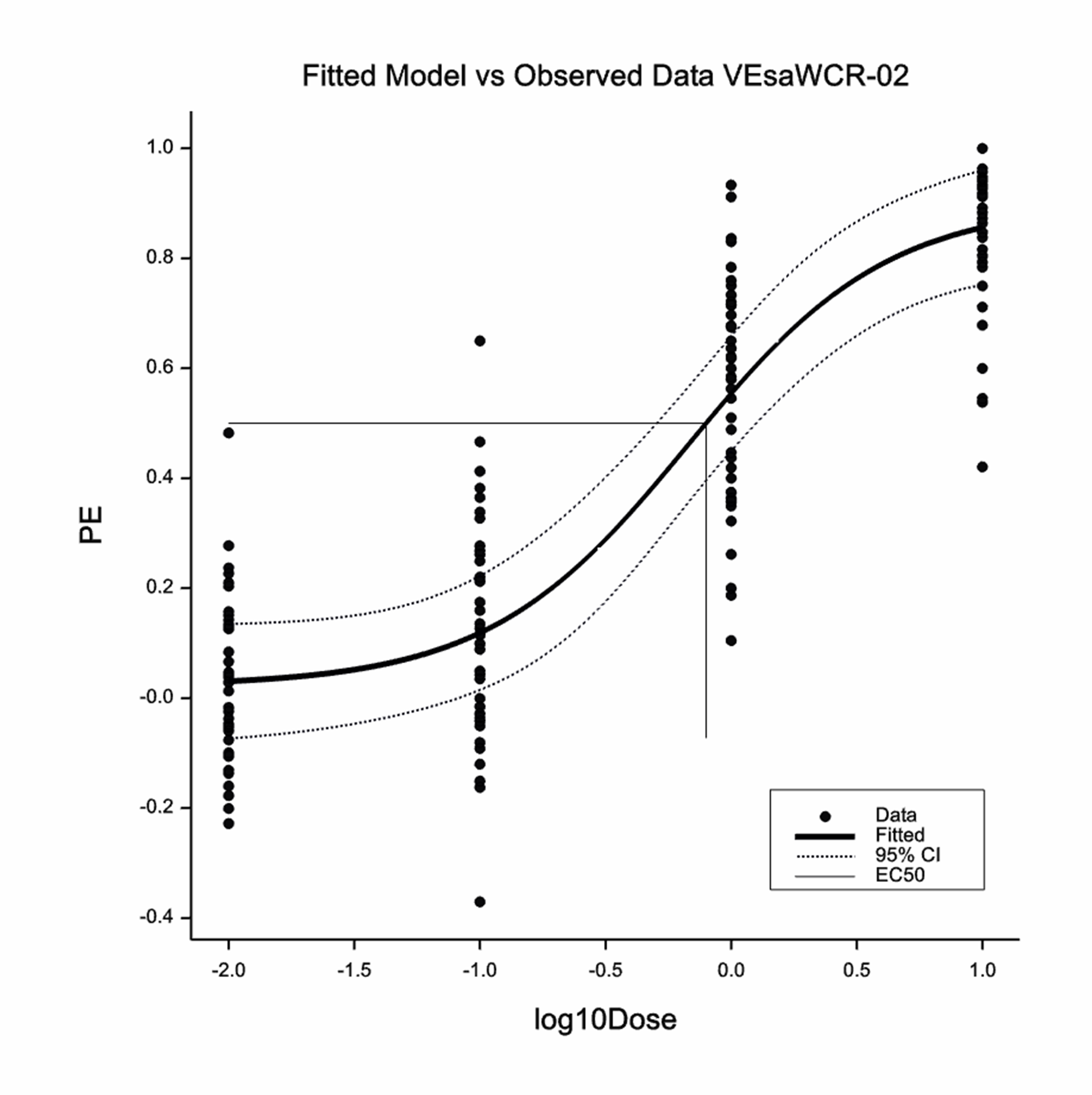

Supplement: Supplementary Figures S1 — The fitted model for each oil, the observed data, the 95% confidence interval around the fitted model and the estimated EC50 are shown in Supplementary Figures; S1, ATbyW02L; S2, ATnaW02B; S3, ATsaW13B; S4, VEbgW01E; S5, LMmeW02H; S6, LTcuW24E; S7, VEbgW01E; S8, VEboW02E; S9, VEbyW06B; S10, VenaW02B; S11, VEsaWCR-01; S12, VEsaWCR-02. (ZIP) [file pone.0048698.s001.zip › Fig S12.tif]
